# Recognizing Moral Identity as a Cultural Construct

**DOI:** 10.3389/fpsyg.2017.00412

**Published:** 2017-03-21

**Authors:** Fanli Jia, Tobias Krettenauer

**Affiliations:** ^1^Department of Psychology, Seton Hall University, South OrangeNJ, USA; ^2^Department of Psychology, Wilfrid Laurier University, WaterlooON, Canada

**Keywords:** identity, culture, Western and Eastern, morality, social context

## Abstract

Current research on moral identity shows that moral identity predicts moral action in Western cultures but not in non-Western cultures. The present paper argues that this may be due to the fact that the concept of moral identity is culturally biased. In order to remedy this situation, we argue that researchers should broaden their scopes of inquiry by adding a cultural lens to their studies of moral identity. This change is important because although some concept of moral identity likely exists in all cultures, it may function in different ways and at different levels in each place. We propose that moral identity is a context-dependent construct tied to varying social and cultural obligations. We argue that Western moral identity stresses an individually oriented morality, whereas, people from Eastern cultures consider a highly moral person to be societally oriented. We conclude by discussing the implications of this view for future research.

## Introduction

For centuries, psychologists, anthropologists, sociologists, and philosophers have tried to explain why people act morally. Kohlberg’s (1969) stage theory of moral development served this task for decades by investigating how moral reasoning influences moral behaviors in hypothetical situations. While Kohlberg’s theory provided insight about the development of moral reasoning skills, his theory is limited because moral reasoning alone is not a strong predictor of moral action (e.g., Blasi, 1983). In an attempt to improve our understanding of why people act morally, researchers have taken on a new approach to moral psychology, which attempts to find a link between moral judgment and moral action. This new approach has raised an interest in the topic of moral identity, which Hardy and Carlo define as “the degree to which being a moral person is important to an individual’s identity” (Hardy and Carlo, 2005, p. 212). Studies have demonstrated that people do appear to form moral identities and that internalizing one’s moral identity can influence moral action (e.g., Krettenauer et al., 2016). While this line of research is promising, some researchers have questioned whether moral identity actually motivates one to act in a moral fashion in non-Western cultures. However, this is problematic because cultural psychologists suggest that people both within and across cultures have different self-concepts, cognitive processes, emotional expectations, and value orientations (Krettenauer and Jia, 2013). Therefore, this paper seeks to address this question by suggesting a culturally inclusive approach to investigating moral identity. First, we will review cultural criticism on moral psychology in general. Next, we will discuss a cultural framework to study moral identity.

## Moral Psychology and Cultural Criticism

Lawrence Kohlberg’s work has heavily influenced the development of moral psychology. His model of moral reasoning and judgment is based, in part, on Piaget’s model of cognitive development. Kohlberg’s theory of moral development (Kohlberg, 1969) proposes six universal stages of development of moral reasoning. This sequence begins with children’s focus on avoiding punishment by authority (Stage 1) and potentially ends with an endorsement of universal principles of justices and rights (Stage 6). The findings from a number of cross-cultural studies have suggested that some aspects of Kohlberg’s theory of morality are universal. For example, Gibbs et al. (2007) revisited Kohlberg’s universality claims by reviewing 75 cross-cultural studies conducted in 23 countries. From this investigation, Gibbs et al. (2007) concluded that there is evidence that Kohlberg’s first four stages may be universal.

However, many researchers have raised concerns regarding the failure of these theories to account for the moral concepts of individuals across diverse cultures (e.g., Dien, 1982). These researchers contend that focusing on concepts of justice, fairness, and harm to individuals and excluding concepts of interdependence, social harmony, and the role of cultural socialization in non-Western settings. This concern is grounded in Western philosophical thought and in the cultural milieu in which Kohlberg developed his theory in the Midwestern United States in the 1950s. Although Western notions of individualism may have been appropriate to describe his theory at the time and place, those same notions may not represent universal moral principles applicable to all people of all cultures.

Since Kohlberg’s time, other scholars have proposed different models to describe moral development for a broad range of cultures. Shweder et al. (1997) have outlined a different approach to moral development, which posits three ethics that are central to the moral belief systems in the majority of cultures around the world: autonomy, community, and divinity. This method of differentiating types of morality not only shows different domains of morality, but also gives us insight into cultural variations (Shweder et al., 1997). For example, there is a more pronounced emphasis on the ethic of community in Taiwan than in the United States and a stronger emphasis on the ethic of autonomy in the United States than in Taiwan (Vauclair and Fischer, 2011).

In his article “The new synthesis in moral psychology,” Haidt (2007) expands Shweder’s theory by proposing the Moral Foundations Model. In opposition to rational theories of moral reasoning, he argues that morality is a quick, automatic process that has formed over human evolution. According to Haidt (2007), the five moral foundations are harm, fairness, ingroup, authority, and purity. In an attempt to determine which moral foundation people endorse, several researchers tested the Moral Foundation Model cross-culturally. One study used a cross-cultural sample, which included participants from Eastern cultures (South Asia, East Asia, and Southeast Asia) and participants from Western cultures (US, UK, Canada, and Western Europe). Haidt found that Eastern participants showed stronger concerns about ingroup and purity compared to Western participants and that Eastern participants were also slightly more concerned about authority.

This examination of the moral psychology literature, which includes Kohlberg’s stages of moral reasoning, Shweder’s ethical codes, and Haidt’s moral foundations, suggests that morality is not culturally universal. People around the world may share the same moral foundations ethical codes, and moral reasoning, but there is much disagreement about their relative importance across cultures. This paper applies a cultural approach to studying morality by focusing on how cultures develop specific ways of thinking and foster certain values (Norenzayan and Heine, 2005). This approach recognizes that there may be some basic universal moral principles, but it argues for powerful influences of culture on various aspects of morality.

## Moral Identity and Cultural Inclusiveness

Recent research in moral psychology has suggested that one can obtain a fuller understanding of moral action by considering the role of the self in morality, which is often termed “moral identity.” Hardy and Carlo explain that moral identity refers to “the degree to which being a moral person is important to an individual’s identity” (Hardy and Carlo, 2005, pp. 212). In other words, if individuals feel that moral values such as being honest, compassionate, fair, and generous are central for defining their personal identity, they have a strong moral identity. While research in this area continues to convince people that moral identity, in Western societies, plays an important part in moral functioning, links between moral identity and non-Western culture remain unclear. For example, Hertz and Krettenauer (2016) conducted a meta-analysis to examine the relationship between moral identity and moral action. Their study included 111 articles from a variety of academic journals. In general, they found a positive correlation between moral identity and moral behavior. However, effect sizes varied in those studies. The effect size was much lower in non-Western cultures than in Western cultures. The authors suggest that the low effect size might be due to different conceptualizations of moral identity between cultures or due to the lack of validity of the current moral identity measures in non-Western cultures. These results call the field’s attention to the issue of ‘cultural uniqueness’ and, thus, they stress an urgent need to assess moral identity in non-Western societies as well as Western ones in order to obtain less biased results.

In his book “Identity and The Life Cycle,” Erikson (1980) considers the way in which we study identity development. He proposes that, in addition to considering ego and personal identity, we should also incorporate cultural context. This is because while ego and personal identity are intrapersonal context areas that lead us to consider personal characteristics and sense of self, adding the cultural context helps us to expand our understanding by encouraging us to consider categories such as native language, country of origin, and racial background. Erikson’s concept of identity aims to establish a social-cultural approach that encompasses all elements of the self, which includes the most internal ego conflicts to the individual’s embeddedness in a cultural context (Schwartz, 2001). This organization reflects Erikson’s view that lifespan development occurs at the interface of self and culture. As a result, identity represents a coherent picture that one shows to both oneself and to the outside world. Thus, moral identity research should not only focus on individual levels, but on a cultural level as well. To what extent moral identity is a function of interacting in a specific culture is a major question that has only recently been raised in moral identity research.

Like many other moral constructs, the moral identity concept is rooted in a Western cultural context that stresses an individually oriented morality. Being a moral person results from a desire to be consistent with one’s moral concepts through which individuals are motivated to gain independence from social conventions. In contrast, people from Eastern cultures consider a highly moral person to be societally oriented. In this moral orientation, people tend to define themselves in the context of collectivism and an interdependent self (Markus and Kitayama, 1991). Social relationships and group membership are linked to the motivation to adjust to the demands of others and to maintain harmony within one’s group (Markus and Kitayama, 1991). Being a moral person in Eastern societies may be more reflective of group norms than of an individual’s morality.

Confucianism provides further support of the societally oriented moral system in Eastern cultures. From the perspective of Confucianism, understandings of morality help to socialize individuals by encouraging them to suppress personal desires in social interactions and to eliminate “Xiao Wo,” personal-centered actions, by emphasizing “Da Wo,” societal-centered actions instead (Hwang, 1999). As a consequence of Eastern ideology, a highly moral person, “I,” is transformed into “we” and, consequently, feelings of society within the group are strengthened.

Chinese education system employed Confucian values of effortful and respectful learning (Hwang, 1999). Consequently, for 1000s of years, Chinese citizens were accustomed to giving, obeying, and following authority. Extended families with hierarchical relationships were also important in traditional Chinese society. Moreover, in the contemporary Chinese history, Cultural Revolution swept the nation in 1970s, driving Chinese to “nation-oriented” collectivism (Yao, 2000). A very popular Chinese analogy of this national value states that “Chinese people are like bricks,” which means that all people have the same functions and that they are willing to be assigned throughout the society wherever ‘society’ needs them (Yao, 2000). Thus, Chinese people should attribute national and societal meanings to the concept of a highly moral person, based on the moral ideology that nation is the most basic and important source of collective identity.

## Cultural Approach of Studying Moral Identity

Empirically, Hertz and Krettenauer (2016) note that the majority of moral identity research is based on the Self-Importance of Moral Identity Questionnaire (Aquino and Reed, 2002). This measure provides participants with a list of nine attributes that are characteristic of a highly moral person (*caring, compassionate, fair, friendly, generous, helpful, hardworking, honest, kind*). However, recent research in virtue ethics, character education, and political orientation across different cultures and religious traditions has suggested that these are Western moral values that need to be broadened (Miller, 2007). The description of Western moral values may fail to adequately generalize the values of other non-Western cultures because Western moral values are limited to a Western understanding of morality. For example, face is an interesting value that is of considerable importance in many Eastern societies, although many Westerners do not have a great understanding of it (Ting-Toomey, 1994). In Western terminology, face is conceptualized as an individual’s positioned identity (Hwang, 2006). In Eastern culture, face is considered the social evaluation of one’s moral character, which is the baseline of one’s integrity of personality (Hwang, 2006). According to Confucian ethics, if any one member of the family does something immoral, all family members may suffer from loss of face (Hwang, 2006). In addition, other values such as “culture of honor” (Leung and Cohen, 2011) and “filial piety” (Hwang, 1999) need to be considered as moral values in non-Western cultures. Thus, we suggest that culturally unique moral values need to be generated through a comprehensive study of the variance of cultural-specific moral identity in non-Western cultures.

One practical methodology of getting one-step closer to measure culturally non-biased moral identity is to create a list of culturally inclusive moral values from both Western and Eastern cultures. Frist, participants from each culture (at least two countries) are asked to describe prototypical conceptions of a highly moral person. For example, Walker and Pitts (1998) asked 120 Canadian adults to generate personality characteristics that were seen as descriptive of a highly moral person using a free-listing procedure. The total number of attributes provided by the participants was 1249. Several judgment rules were used to reduce the number of descriptors that participants had listed: any phrases and sentences were divided into single descriptors; adjectives were used instead of nouns; synonymous terms were combined; antonym pairs which were generated less frequently were deleted; idiosyncratic responses were eliminated (Walker and Pitts, 1998). Finally, 92 attributes as descriptive of a highly moral person were included in the study. The similar procedure has also been used in US (Aquino and Reed, 2002; Hardy et al., 2011).

However, prototypical conceptions of a highly moral person in Eastern cultures have been neglected. We suggest first replicating the previous procedure to ask participants from Eastern countries (e.g., China, Korean, India) to free list moral values representing Eastern view of a highly moral person. Second, the Eastern moral values should be revised in accordance with the Walker and Pitts’ (1998) judgment rules. Third, researchers should compare the remaining Eastern moral values with the moral values generated in Western culture. Common descriptors between the two cultural lists should be identified as culturally shared moral values. Unique descriptors between the two cultural lists should be observed as culturally non-shared moral values in each culture. Finally, a joint list of shared and non-shared descriptors exemplifies a culturally inclusive moral values that describes a highly moral person in both Western and Eastern societies. We suggest that researchers should consider this culturally non-biased approach to investigate moral identities in different cultures, although the procedure makes it a time-consuming technique to use.

In a first study (Jia, 2016), we strived to identify value attributes that describe individuals’ prototypical conceptions of a “highly moral person” in Chinese culture. We asked 109 Chinese college students to write down at least 10 attributes of a highly moral person in their points of view. A total of 1,924 attributes were created. In the second step, we applied the Walker and Pitts’ (1998) procedure to reduce the number of attributes. In the third step, we compared the remaining attributes with a list of moral values frequently used from predominantly Western cultures (Krettenauer et al., 2016). We found 17 culturally specific attributes from the Chinese sample: “*peaceful, credible, incorruptible, warm-hearted, motivated, ambitious, diligent, civilized, patriotic, solidaric, careful, prudent, filial piety, dedicated, principled, active, and outgoing.”* In addition, we found 17 unique Western attributes that Chinese participants did not mention in their descriptions of a highly moral person. Those attributes were “*accepting, confident, consistent, educated, follows the rules, fun, good, happy, has high standards, healthy, humble, makes the right choices, non-judgmental, obedient, proper, proud, religious.*” The Chinese list used for defining a moral person was illustrated by specific values that not only reflect upon individually oriented morality such as “*credible* and *warm-hearted*,” but also imply societally oriented morality (e.g., *patriotic* and *prudent*). There are certain concepts such as *filial piety* and *solidaric* that have been mentioned in the literature as specific Eastern values for a long time (Hwang, 1999). Other attributes such as *peaceful, principled, credible*, and *incorruptible* that are consistent with Confucian values of living in harmony with others and societies.

## Conclusion

Although we are certainly not the first to worry about the generalizations of predominant Western theories and methodologies in moral psychology, our efforts to compile theoretical and empirical cases have revealed an alarming situation in research concerning moral identity. Previous findings maybe replicable across multiple samples in Western societies using different methods such as self-report and interview and those methods may be applicable to non-Western society; however, researchers must also investigate the levels and degrees to which the concept of moral identity can be accessible across cultures (Henrich et al., 2010). Thus, we conclude: (1) Conceptually, moral identity consists of both individual and societal orientations; (2) Empirically, the study of moral identity requires a culturally non-biased methodological tool.

## Author Contributions

FJ conceptualized the viewpoint and wrote the complete draft. TK supervised FJ’s Ph.D. dissertation and provided critical feedback in this research.

## Conflict of Interest Statement

The authors declare that the research was conducted in the absence of any commercial or financial relationships that could be construed as a potential conflict of interest.

## References

[B1] Aquino K. Reed A. II. (2002). The self-importance of moral identity. *J. Pers. Soc. Psychol.* 83 1423–1440. 10.1037/0022-3514.83.6.1423 12500822

[B2] Blasi A. (1983). Moral cognition and moral action: a theoretical perspective. *Dev. Rev.* 3 178–210. 10.1016/0273-2297(83)90029-1

[B3] Dien D. S. F. (1982). A Chinese perspective on Kohlberg’s theory of moral development. *Dev. Rev.* 2 331–341. 10.1016/0273-2297(82)90017-X

[B4] Erikson E. H. (1980). *Identity and the Life Cycle: A Reissue.* New York, NY: Norton.

[B5] Gibbs J. C. Basinger K. S. Grime R. L. Snarey J. R. (2007). Moral judgment development across cultures: revisiting Kohlberg’s universality claims. *Dev. Rev.* 27 443–500. 10.1016/j.dr.2007.04.001

[B6] Haidt J. (2007). The new synthesis in moral psychology. *Science* 316 998–1002. 10.1126/science.1137651 17510357

[B7] Hardy S. A. Carlo G. (2005). Identity as a source of moral motivation. *Hum. Dev.* 48 232–256. 10.1159/000086859

[B8] Hardy S. A. Walker L. J. Olsen J. A. Skalski J. E. Basinger J. C. (2011). Adolescent naturalistic conceptions of moral maturity. *Soc. Dev.* 20 562–586. 10.1111/j.1467-9507.2010.00590.x

[B9] Henrich J. Heine S. J. Norenzayan A. (2010). The weirdest people in the world? *Behav. Brain Sci.* 33 61–83. 10.1017/S0140525X0999152X 20550733

[B10] Hertz S. G. Krettenauer T. (2016). Does moral identity effectively predict moral behavior?: a meta-analysis. *Rev. Gen. Psychol.* 20 129–140. 10.1037/gpr0000062

[B11] Hwang K. (1999). Filial piety and loyalty: two types of social identification in Confucianism. *Asian J. Soc. Psychol.* 2 163–183. 10.1111/1467-839X.00031

[B12] Hwang K. K. (2006). Moral face and social face: contingent self-esteem in Confucian society. *Int. J. Psychol.* 41 276–281. 10.1080/00207590544000040

[B13] Jia F. (2016). *Moral Identity from Cross- and Bi-Cultural Perspectives* (unpublished doctoral dissertation). Wilfrid Laurier University Waterloo, ON.

[B14] Kohlberg L. (1969). *Stage and Sequence: The Cognitive-Developmental Approach to Socialization.* New York, NY: Rand McNally.

[B15] Krettenauer T. Jia F. (2013). Investigating the actor effect in moral emotion expectancies across cultures: a comparison of Chinese and Canadian adolescents. *Br. J. Dev. Psychol.* 31 249–362. 10.1111/bjdp.12012 23901847

[B16] Krettenauer T. Murua L. A. Jia F. (2016). Age-related differences in moral identity across adulthood. *Dev. Psychol.* 52 972–984. 10.1037/dev0000127 27124654

[B17] Leung A. K. Y. Cohen D. (2011). Within-and between-culture variation: individual differences, and the cultural logics of honor, face, and dignity cultures. *J. Pers. Soc. Psychol.* 100 507–526. 10.1037/a0022151 21244179

[B18] Markus H. R. Kitayama S. (1991). Culture and the self: implications for cognition, emotion, and motivation. *Psychol. Rev.* 98 224–253. 10.1037/0033-295X.98.2.224

[B19] Miller J. G. (2007). “Cultural psychology of moral development,” in *Handbook of Cultural Psychology* eds Kitayama S. Cohen D. (New York, NY: Guilford Press) 477–499.

[B20] Norenzayan A. Heine S. J. (2005). Psychological universals: what are they and how can we know? *Psychol. Bull.* 131 763–784. 10.1037/0033-2909.131.5.763 16187859

[B21] Schwartz S. J. (2001). The evolution of Eriksonian and, neo-Eriksonian identity theory and research: a review and integration. *Identity* 1 7–58. 10.1207/S1532706XSCHWARTZ

[B22] Shweder R. Much N. Mahapatra M. Park L. (1997). “The “big three” of morality (autonomy, community, divinity), and the “big three” explanations of suffering,” in *Morality and Health* eds Brandt A. Rozin P. (New York, NY: Routledge).

[B23] Ting-Toomey S. (1994). *The Challenge of Facework: Cross-Cultural and Interpersonal Issues.* Albany, NY: SUNY Press.

[B24] Vauclair C. M. Fischer R. (2011). Do cultural values predict individuals’ moral attitudes? A cross-cultural multilevel approach. *Eur. J. Soc. Psychol.* 41 645–657. 10.1002/ejsp.794

[B25] Walker L. J. Pitts R. C. (1998). Naturalistic conceptions of moral maturity. *Dev. Psychol.* 34 403–419. 10.1037/0012-1649.34.3.403 9597350

[B26] Yao X. (2000). *An Introduction to Confucianism.* Cambridge: Cambridge University Press 10.1017/CBO9780511800887

